# Addressing career readiness of allergy and immunology fellows in training

**DOI:** 10.1016/j.jacig.2025.100504

**Published:** 2025-06-03

**Authors:** Maureen Bauer, Cullen Dutmer, William C. Anderson, Dan Atkins

**Affiliations:** Children’s Hospital Colorado, University of Colorado School of Medicine, Section of Pediatric Allergy and Immunology, Aurora, Colo

**Keywords:** Career planning, allergy and immunology fellowship

## Abstract

**Background:**

Career readiness has been identified as a significant unmet need in allergy and immunology fellowship training programs despite 90% of graduates attending career development sessions provided by local or national societities.

**Objective:**

Our aim was to develop and implement a career readiness curriculum for allergy and immunology fellows and assess its effectiveness.

**Methods:**

A 6-part career readiness curriculum providing instruction in previously identified areas of deficit was developed in 2021. All sessions were conducted either as individual sessions between the trainee and core faculty or in a small group setting, allowing the curriculum to be customizable for each individual. Quantitative and qualitative assessments were utilized to evaluate the success of the curriculum. Trainees’ comfort and knowledge were assessed via a 7-point Likert scale before and after implementation of the curriculum. To obtain additional feedback, qualitative interviews were conducted with members of each of the first 3 cohorts in which the curriculum was used.

**Results:**

Of the 6 trainees who were eligible to participate between 2021 and 2024, all 6 (100%) participated. The trainees’ comfort and knowledge, which were assessed via a 7-point Likert scale, improved after completion of the currkculum. Qualitiative interviews revealed that the curriculums success was due to its customizable nature, which provided individualized feedback. The highest-yield session, as recognized by the trainees, was a discussion panel with recent graduates. It was a supplement to the resources available in other settings. Implementation of a career readiness curriculum contributed to high fellow satisfaction on the annual Accreditation Council for Graduate Medical Education survey.

**Conclusions:**

Individualized career planning should be regularly incorporated into graduate medical education training.

## Introduction

Career development is not often formalized in residency or fellowship training programs. A qualitative assessment of 89 internal medicine residents noted that 75% found career planning at least somewhat stressful, with most believing that career planning should be incorporated into the curriculum and occur early in residency.^1^ The limited literature on the topic of career planning demonstrates benefit with dedicated education. An evaluation of a 1-year career development course offered to fellows in geriatric medicine, dentistry, and psychiatry at the University of Rochester noted that 100% of participants (9 in total) believed that the course positively affected their career planning.^2^ Similarly, a career development symposium for interventional pulmonary fellows was associated with improved knowledge and skills, with 84% of participants finding it beneficial.^3^ Despite the success of prior programs, there is an overall paucity of literature on the topic, with no prior publications addressing career readiness in allergy and immunology (A&I) fellowship training programs.

Recent national data from a study of Accreditation Council for Graduate Medical Education (ACGME)-accredited fellowship graduates in A&I noted that 40% of trainees had difficulty obtaining a position after graduation, with 77% of individuals applying for more than 4 positions.^4^ A prior gap analysis and targeted needs assessment of A&I fellowship graduates at Children’s Hospital Colorado identified career readiness (defined as identifying career interests and acquisition of the knowledge and skills to find a suitable position) to be a more pressing unmet need than perceived deficits in medical knowledge or procedural competency.^5^ Notably, this deficit occurred despite 90% of graduates having attended career development sessions provided by local or national societies. Herein we describe the development, implementation, and assessment of a career readiness curriculum for A&I fellows at Children’s Hospital Colorado.

A total of 8 topics within career readiness were previously identified as not being routinely addressed in other settings.^2^ Briefly, thise prior work, which guided the development of a 6-session career readiness curriculum, included a targeted needs assessment performed using a modified Delphi method that entailed interviews with prior and current fellows to identify a comprehensive list of career planning insufficiencies. Participating fellows subsequently ranked deficits by perceived significance and/or impact to identify the following 8 topics of maximum importance to be addressed at the program level: (1) networking, (2) timeline for a job search, (3) payment and reimbursement structures, (4) career paths within private practice and academic medicine, (5) contract negotiations, (6) key questions and/or red flags, (7) curriculum vitae (CV) and cover letter development, and (8) interview preparedness. Given the overlap within these topics, 6 sessions were developed to address the unmet needs.

The sessions were conducted either on an individual basis with the trainee and faculty member(s) or in a small group setting with all trainees and select faculty (3-8 individuals in total). This curriculum began midway through the first year of fellowship and ended at the beginning of the second year of fellowship to ensure completion before initiation of the job search process for a 2-year fellowship. Thus, the 6 sessions occured over a 9-month period. A detailed description of each session follows.

### Session 1: Networking skills and job application timeline

Format: Small group session with all trainees and 4 faculty members.

Duration: 1 hour.

Content: A suggested timeline for the job search process was developed and presented by the training program director on the basis of feedback from prior graduates that was obtained during the targeted needs assessment. In addition, a presentation on networking that included definitions, examples of a network, and benefits of networking was provided and followed by a small group discussion of sample networking e-mails and experiences.

### Session 2: CV and cover letter development

Format: Individualized feedback session with each trainee and 4 faculty members.

Duration: 30 minutes with each trainee.

Content: Before the session, sample CVs and cover letters were sent to trainees as suggested templates. Trainees sent their individual CV and cover letter(s) to faculty members before the session for review. Each trainee had a dedicated session during which faculty members provided individualized feedback and identified areas for improvement.

### Session 3: Academic medicine career paths

Format: Small group discussion that included the A&I section head at Children’s Hospital Colorado and all trainees without formal presentation.

Duration: 1 hour.

Content: The section head led an open-ended discussion that included the following suggested topics: payment and reimbursement structures, contract negotiations, key questions to ask in interviews, and career paths within academic medicine.

### Session 4: Private practice career paths: Question and answer (Q&A) session with community and former graduates

Format: Small group discussion (virtual) without formal presentation.

Duration: 1 to 2 hours.

Content: In the first year of curriculum implementation, we partnered with a local allergy society to identify physicians in private practice to serve on a panel discussion with our trainees and other trainees in the surrounding area. Although this provided a range of opinions, given the larger group setting, there was less candor and less individualized feedback provided to trainees. Therefore, we altered the format to have 2 separate Q&A sessions with our prior graduates (one for those in academic positions and the other for those in private practice). These were open-ended discussions, with suggested topics provided before the session. To allow for a candid conversation, program leadership was not present at these sessions. Suggested topics for discussion included salary and reimbursement structures, partner opportunities, contract negotiations, clinical expectations, and key questions to ask regarding private practice positions.

### Session 5: Academic career paths: Q&A with prior graduates

Format: Small group discussion (virtual) without formal presentation.

Duration: 1 to 2 hours.

Content: Following the first-year feedback, a separate session with prior graduates currently in academic medicine was provided. This was an open-ended conversation with suggested topics provided before the session. To allow for a candid conversation, program leadership was not present at these sessions. This session was in addition to the academic medicine career path session with the section head, as trainees believed thatg both were informative and offered differing perspectives. Suggested topics for discussion included payment models, contract negotiations, key questions to ask, and career paths within academic medicine.

### Session 6: Interview preparedness

Format: Simulated interviews with individualized feedback.

Duration: 1 hour (30 minutes per each of 2 mock interviews).

Content: Each trainee completed 2 mock interviews, one simulating a private practice position and the other an academic position. Each mock interview had 1 faculty member simulating the interviewer and a second faculty member observing to provide individual feedback. Mock interviews were based on real job postings and/or tailored to specific positions that fellows were pursuing, which were provided to the faculty beforehand.

Quantitative and qualitative assessment was utilized to evaluate the efficacy of the curriculum over the first 3 cohorts of trainees. A quantitative survey was administered to assess trainee comfort and knowledge in deficits within career readiness before and after completion of the curriculum that were previously identified by using a 7-point Likert scale. A lower response corresponds with a higher level of comfort and/or knowledge in the area. A 2-sample *t* test was used to evaluate for statistical significance. This curriculum was determined by the University of Colorado institutional review board to be exempt/non-human subject research.

## Results and discussion

**Fig 1:**
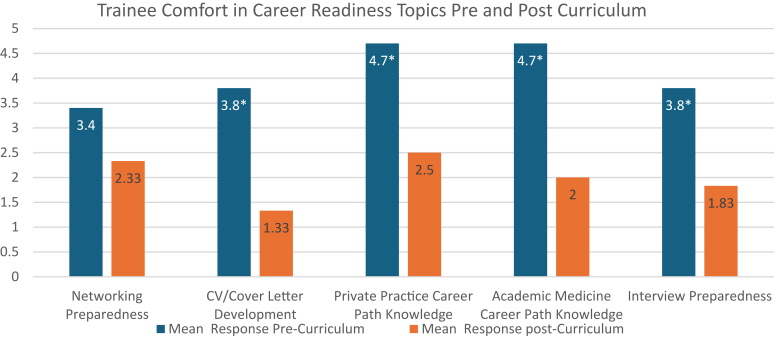
Trainees were assessed regarding their comfort and knowledge in the previously identified areas of deficit before (*blue*) and after (*orange*) completion of the curriculum via a 7-point Likert scale, with lower scores indicating higher comfort and/or knowledge in the area. ∗*P* < .05.

Between 2021 and 2024, 6 trainees were eligible to participate (2 trainees per academic year), with all 6 participating (a 100% participation rate). Quantative assessment demonstrated an improvement in trainee comfort and knowledge after curriculum completion (Fig 1). More specifically, after implementation of the curriculum there was a trend toward lower mean scores (higher knowledge of and/or comfort regarding the topic) in all categories, with statistical significance achieved in 4 of the 5 (*P* < .05). Networking preparedness had the least improvement in knowledge and/or comfort, indicating an area for ongoing revision.

Qualitative feedback was solicited via open-ended interviews with each cohort of trainees. A major critique identified in the first year of the curriculum was the format of the private practice session. Specifically, the panel of private practice physicians in the community was thought to be the least beneficial session, secondary to a longer (>3 hours) and less candid conversation with advice being less individualized. Trainees believed that information discussed in that session was covered in other venues. As the Q&A with our prior graduates was the most beneficial session according to qualitative feedback, trainees suggested having 2 Q&A sessions, one with graduates in academic and the other with physicians in private practice, which we then implemented with improved qualitative feedback in subsequent years.

The qualitative feedback additionally revealed that although similar sessions attended through local or national societies provided a strong base of general knowledge, the success of this curriculum was due to it being customizable and providing individualized feedback. Sample quotes from interviews include, “I felt highly prepared for interviews. Questions that I received feedback on during my mock interviews were asked and I was more prepared in my response.” and “This curriculum was beneficial because it was customized to each trainee. It complemented the broader resources available in other settings.”

Trainees believed that although all of the sessions were informative, the most valuable session was the Q&A with our prior graduates. As those individuals had most recently completed the job search process, they were able to provide valuable advice that was not readily available elsewhere.

Although career readiness is not directly addressed in the annual ACGME trainee survey, development of this curriculum was associated with an improvement in the mean overall trainee evaluation of our fellowship program, with our program consistently rated higher than the national mean score after implementation (Table I). Although there are likely many factors that affect this score, the addition of this curriculum likely contributed to this positive trend.

**Table I:** Trainees’ mean overall evaluation of the program on the ACGME survey

Academic year	Program mean	National mean
2017-2018	4.2	4.4
2018-2019	4.5	4.4
2019-2020	4.5	NA∗
2020-2021	4.8	4.3
2021-2022†	5.0	4.3
2022-2023	5.0	4.3
2023-2024	5.0	4.2

*NA*, Not available.

∗ The ACGME did not provide national mean comparison scores because of the coronavirus 2019 disease (COVID-2019) pandemic.

† The year during which the career readiness curriculum was implemented.

Our career readiness curriculum for A&I fellows was successful, as evidenced by an improved understanding of and knowledge on core topics within career planning following completion of the curriculum. Qualitative feedback indicated that the success of the curriculum was largely due to its personalized nature, providing trainees individual feedback as a supplement to the general information provided in larger group settings. Other publications on career planning curricula have also included individual or small group sessions, indicating that this is likely an essential component for success.^2^^,^^3^

Notably, the session that was consistently reported as being the most informative was our Q&A session with prior graduates, given their unique insight as individuals who had recently completed the process. This session would be feasible for other training programs to implement locally. Additionally, although partnering with a local allergy society was not effective for our program, it may be a more successful option for programs in regions without multiple training programs.

Although not a direct assessment of the curriculum, our program had improved trainee evaluation of the overall program on the annual ACGME survey after implementation, which reflects at least partly the impact of the curriculum. Thus, other fellowship training programs may want to implement a similar curriculum. The sessions listed in this report would be reasonable for a training program director to develop and implement, as it requires only a small number of motivated faculty. It would also be beneficial if specific questions on career readiness were included on the American Academy of Allergy, Asthma & Immunology fellow annual exit survey to better define the need on a national level.

The limitations of this assessment include the small number of participants, as a result of which results may not be generalizable. Additionally, the small size our of training program, which lended itself well to individualized and small group settings, may be difficult to implement in larger programs. Moreover, the ACGME annual survey does not specifically address career readiness; thus, use of this surevey as a measure is not a direct evaluation of the curriculum. A change in program leadership and other educational changes occurred during that time, which also affected results. However, the curriculum likely contributed, in part, to the assessment’s positive findings.

Our career readiness curriculum is likely applicable to other A&I programs, as well as to other residency and fellowship programs, as many elements were not specific to A&I. Individualized career planning should be regularly incorporated into graduate medical education training.

**Key messages**

• Career readiness has been identified as an unmet need in A&I training programs.
• Individualized and customizable sessions for trainees at the programm level are a complement to more general career planning sessions offered in larger group settings.
• Discussion panels with recent graduates in the field is recognized by trainees as a high-yield experience.

## Disclosure statement

Disclosure of potential conflict of interest: W. C. Anderson has served on advisory boards for Regeneron and Sanofi. He has received program development grants from Colorado Medicaid and the 10.13039/100011857COPIC Medical Foundation. M. Bauer has served on advisory boards for Regeneron, Sanofi and DBV. She has received consulting fees from Dynamed. C. Dutmer has served on advisory boards for AMGEN Inc. and Pharming Pharmaceuticals. The remaining author declares that he has no relevant conflicts of interest.
